# Fiber Bragg Grating Embedded 3D-Printed Insole with Commercial and Portable Reader for Stance Phase Determination

**DOI:** 10.3390/bios15090623

**Published:** 2025-09-19

**Authors:** Arnaldo Leal-Junior, Mariana Silveira, Jan Nedoma, Radek Martinek

**Affiliations:** 1 Graduate Program in Electrical Engineering, Federal University of Espírito Santo, Vitória 29075-910, Brazil; mariana.silveira@ufes.br; 2 Department of Cybernetics and Biomedical Engineering, VSB—Technical University of Ostrava, 70800 Ostrava, Czech Republic; radek.martinek@vsb.cz; 3 Department of Telecommunications, Faculty of Electrical Engineering and Computer Science, VSB—Technical University of Ostrava, 70800 Ostrava, Czech Republic; jan.nedoma@vsb.cz

**Keywords:** fiber bragg gratings, instrumented insoles, ground reaction forces, wearable sensing

## Abstract

This paper presents development and application of a Fiber Bragg Grating (FBG) array embedded in a 3D-printed insole for ground reaction force (GRF) estimation. In this case, a 3D-printed insole is fabricated from a scanned commercial insole in which a 5-FBGs array is integrated. The FBGs are characterized as a function of the applied transverse force, where a mean sensitivity of 0.11 ± 0.10 pm/N was obtained considering all FBGs. A portable FBG signal acquisition system was connected to the FBG array embedded in the insole and tested for the GRF analysis in a healthy volunteer. The gait tests results indicate stance and swing phases of 41.0 ± 6.5% and 59 ± 6.5%, respectively, which are within reference values of the literature. Furthermore, a 0.904 R^2^ was found in the correlation analysis of the measured GRF response and the conventional M-shaped curve for the GRF in which all subdivisions of the stance phase were detected.

## 1. Introduction

Optical fiber sensors, an emerging sensor technology, present numerous advantages over conventional electronic sensors, including lightweight design, compact size, chemical stability, immunity to electromagnetic interference, and the multiplexing capabilities [1]. The widespread use of optical fiber sensors has coincided with the development of various interrogation techniques and sensor design approaches [2].

Nowadays, there is a wide range of optical fiber sensor systems, including those based on intensity variation [3], optical interferometry [4], nonlinear effects [5], fiber specklegrams [6], surface plasmon resonance [7], as well as fiber Bragg gratings (FBGs) [8]. These diverse optical fiber sensors have been applied to measure numerous physical and chemical parameters, with each approach offering distinct advantages and limitations. Sensors such as optical interferometers, LPGs (Long Period Gratings), and FBGs measure the target parameter through changes in wavelength, making them insensitive to fluctuations in optical source power [9]. Additionally, these sensors generally offer higher accuracy and resolution compared to intensity-based sensors. FBGs, in particular, provide the added benefit of excellent multiplexing capabilities, allowing multiple quasi-distributed sensor arrays to be inscribed at different Bragg wavelengths within a single fiber [10]. Furthermore, their small dimensions and flexibility make it possible to integrate them into different materials [11], enabling the development of functional structures such as instrumented devices for wearable applications [12] and smart textiles [13] with many applications in gait analysis as well as smart components and neuropsychological assessment using finger tapping tests [14]. Moreover, the use of FBGs in biomedical applications has been thoroughly explored in the last few years [15,16].

Following one of the previously mentioned optical fiber sensors applications, gait analysis involves the evaluation of kinematic and kinetic parameters, which provide valuable information for clinicians in selecting the appropriate treatment for gait-related pathologies [17]. The gait cycle is defined as the interval between two consecutive, identical events in human gait, typically starting with the initial contact of one foot and ending when the same foot touches the ground again. The cycle is divided into two main phases: stance and swing [18]. The stance phase usually accounts for 62% of the gait cycle [19], although this percentage can vary depending on gait velocity. During the stance phase, the ground reaction force (GRF), which is equal and opposite in direction to the body’s motion, changes in both direction and magnitude as the body’s center of mass moves forward while the foot remains in contact with the ground [20]. Since the GRF varies throughout the stance phase of the gait cycle, it can be used to assess this phase. As a result, the GRF is a useful tool for distinguishing between normal and pathological gait [21] and can also help predict the risk of ulceration in certain conditions [22].

The aforementioned advantages make optical fiber sensor technology (mainly FBG sensing approach) suitable for plantar pressure measurement. Liang et al. [23] used FBG sensors in a plantar pressure detection system for static measurements. A similar setup was presented by Suresh et al. [24], who developed an insole for measuring pressure under different postural conditions. Domingues et al. [25] introduced an insole with FBG sensors for both static and dynamic measurements of GRF. However, wearable systems for dynamic monitoring of human activities present a unique challenge, as they often require a combination of the limitations typically associated with conventional interrogation systems for wavelength-based sensors. These systems need to be portable with a high acquisition rate and must be capable of measuring numerous sensors simultaneously, as monitoring human activities typically involves tracking multiple physical parameters, such as joint angles, physiological signs, and force and torque interactions [26]. Additionally, a low-cost system is highly desirable, especially for e-health solutions that enable patients to monitor their health conditions or activities from home [27]. Therefore, the low cost is also a key requirement for wearable systems in healthcare or human activity monitoring. Considering specific development in optical fiber instrumented insoles, the development of intensity variation-based insoles was proposed in [28]. However, the lack of multiplexing capabilities makes FBG-based insole such as the ones proposed in [29] a suitable option for ground reaction forces analysis. However, in FBG applications, commercial FBG interrogators are often used, resulting in bulky devices that hinder the portability of the sensor system [30]. As one of the methods for a portable and lower cost FBG sensor development, the edge filtering technique is based on the frequency-to-amplitude conversion of wavelength-based optical fiber sensors in which the spectral variations in an FBG are directly translated into changes in optical power [31]. This method relies on the convolution in the time domain of the spectra from the FBG sensor and the edge filter, i.e., another optical filter that can be achieved using different approaches, such as a second FBG [32] or an interferometer [31]. Considering this background, Table 1 presents the comparison between the proposed instrumented insole and the previously proposed insoles in the literature.

**Figure 1:**
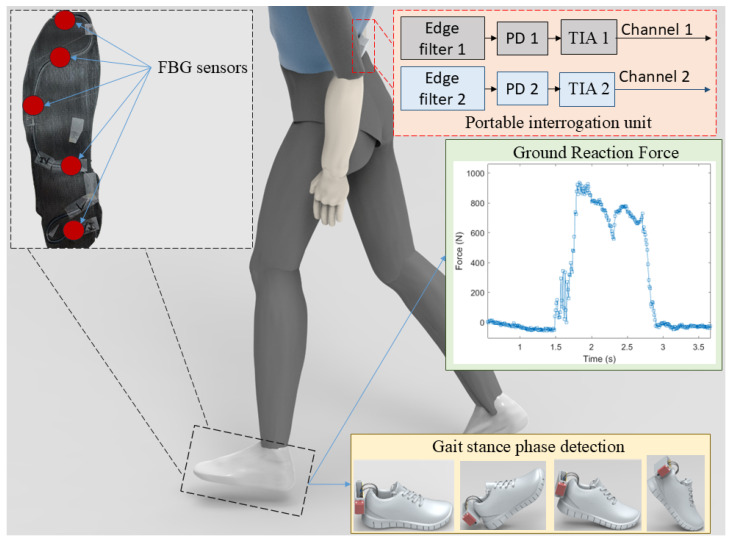
Schematic representation of the FBG-embedded insole principle and potential applications. PD: photodetector; TIA: Transimpedance Amplifier.

This paper presents development and application of an FBG-embedded insole for force distribution and GRF estimation. The proposed instrumented insole combines the sensitivity, multiplexing capabilities and accuracy of FBG approach with the low cost and portability of the intensity variation analysis by means of using two edge filters in conjunction with the FBG array embedded in the insole. Thus, considering the contributions of this work, such new approach for FBG-based instrumented insoles provide a higher portability than the previously reported ones using FBGs [35], whereas the proposed solution provides a more stable, accurate and higher resolution detection than the insoles based on intensity variation sensors [28]. In addition, the proposed sensor system not only can provide real-time estimation of the GRF during gait but also leads to the assessment of the subdivisions of the gait, which has important implications in gait analysis and evaluation of locomotor disorders. The low cost of the proposed system can make it available to remote health monitoring of patients as well as the continuous gait monitoring to evaluate fall risk and physical rehabilitation in different groups of patients. In order to summarize the paper contributions, Figure 1 presents a schematic overview of the proposed insole functionalities and applications.

## 2. Materials and Methods

### 2.1. Prototype Description

**Figure 2:**
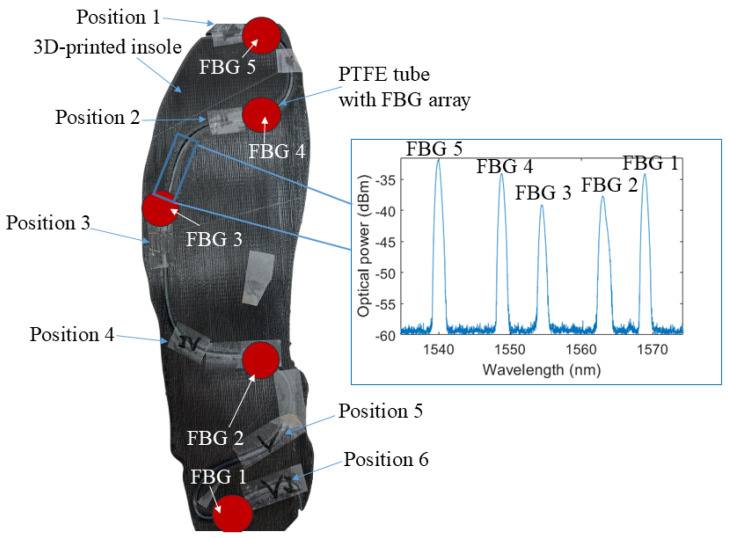
Schematic representation of the FBG-embedded insole and the positioning for the characterization tests. Figure inset shows the reflected spectrum of the FBG array.

As the first step on the proposed FBG-embedded insole, the FBG array is inscribed using a Nd: YAG laser (CNI Laser DPS-266-Q, Changchun, China) with operating wavelength of 266 nm and nanosecond pulses of 8 ns. The focal lens (LJ4862-UV, Thorlabs, São Carlos, Brazil) is used to focus the laser beam on the phase masks (5 different phase masks were used to inscribed the 5-FBG array). The phase mask is positioned on the focal distance of the lens using two 3D translation stages (MBT602/M, Thorlabs, São Carlos, Brazil). The optical fiber used is a conventional Boron-doped photosensitive single mode fiber (PS1250/1500, Fibercore, Campinas, Brazil). The FBG array includes 5 FBGs positioned on the regions close to the ones with the highest pressure during gait (as discussed in [36]), where the distance between FBGs and their positioning in the insole are presented in Figure 2. All the FBGs have a physical length of around 10 mm and the distance between each grating is around 5 cm. It is important to mention that the FBG length is not sufficiently high to create chirping effects or significant birefringence changes on the grating when the transverse pressures are applied, since the pressure distribution in such small regions of the foot (i.e., 10 mm) does not lead to significant pressure differences along the FBG region.

The insole for FBG embedment is manufactured via additive manufacturing using an Ultimaker S5 3D printer (Ultimaker, Zaltbommel, The Netherlands) and Thermoplastic Polyurethane (TPU) filament, processed with 100% infill density to ensure mechanical stability and durability. For the insole model design, a commercial insole was adopted as a geometric reference. A 3D scanner (Sethi3D, Campinas, Brazil) was employed to acquire the digital geometry of the reference insole. Following the 3D scan, the virtual model was modified to incorporate a longitudinal groove, as illustrated in Figure 2, designed to house the optical fiber. The groove was integrated into the 3D-printing process of the TPU insole structure. The optical fiber containing the inscribed FBG array is positioned inside a capillary tube fabricated from PolyTetraFluoroEthylene (PTFE). To enhance structural robustness and ensure effective strain transfer from the insole to the FBG sensing regions, the PTFE tube is filled with a PolyDiMethylSiloxane (PDMS) resin. The resin not only improves the mechanical coupling between the host structure and the optical fiber but also increases long-term stability, robustness, and repeatability of strain transmission under applied loading conditions. After encapsulation, the PTFE tube containing the FBG array and PDMS is maintained at ambient room temperature (26 °C) for 24 h to allow complete curing of the PDMS. Finally, the encapsulated PTFE tube is inserted into the groove of the 3D-printed insole. Figure 2 provides a schematic overview of the instrumented insole, highlighting the integration of the FBG array and the corresponding reflected spectrum of the sensing elements.

**Figure 3:**
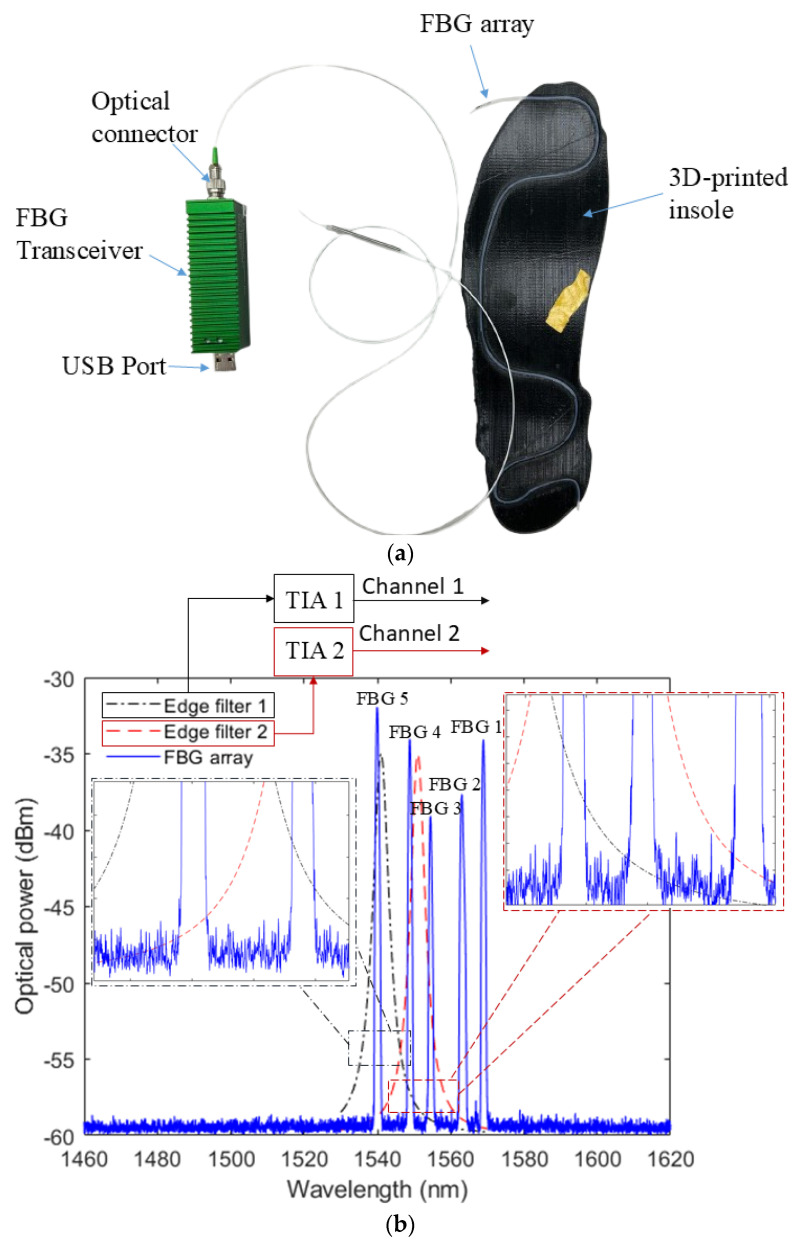
(**a**) Photograph of the signal acquisition system connected to the insole. (**b**) Schematic representation of the edge filtering used in the FBG-embedded insole.

The prototype is represented in Figure 3a, where it is possible to observe the FBG array connected to the FBG portable transceiver (FBGT200, Redondo Optics, Redondo Beach, CA, USA), which has a size of 25 mm × 25 mm × 76 mm and a mass of 70 g. The system includes two transimpedance amplifiers and two filters, connected to two photodetectors. In addition, the system is capable of a maximum wavelength resolution of 10 pm. The voltage variation for each channel is collected and, for signal processing, it can be transmitted to a PC (for the comparison with bulk interrogator) or microcontroller through the USB port of the device to provide a fully portable system. To enhance the portability, the acquisition system can be connected to Raspberry Pi 5 with around 45 g in which the data is stored using an SD card, since all data processing is performed offline. The signal processing relies solely on the calibration-derived polynomial, enabling direct implementation within the microcontroller. Considering an option without the computer connection, the complete system exhibits a power consumption of approximately 2000 mA. Powered by a 10,000 mAh/37 Wh external battery pack (220 g), the setup provides an operational autonomy of 3 h.

For initial characterization, the insole was connected to a commercial FBG interrogator (Hyperion si255, Luna Inc., Roanoke, VA, USA), enabling high-accuracy wavelength analysis of each grating. However, the lack of portability of such systems limits their suitability for wearable applications. Therefore, following the baseline characterization, the FBG-embedded insole was evaluated using a portable interrogation unit based on the edge-filter technique. The system employs two edge filters, each coupled to a photodetector with a transimpedance amplifier (TIA) for optical power acquisition. The convolution of the FBG array response with the transmission profiles of the filters is recorded at 100 Hz sampling frequency. Figure 3b illustrates the principle of operation, where the output of each edge-filter corresponds to the superposition of the contributions from all FBGs spectrally aligned with the respective filter.

### 2.2. Experimental Analysis

For force characterization, loads ranging from 20 N to 1000 N were applied at six distinct regions of the instrumented insole, as indicated in Figure 2. Each test was repeated three times, yielding three measurement cycles. The selected force range corresponds to the expected loading conditions during gait, while the chosen positions were designed to assess sensitivity both when forces were applied directly over the FBG region and when applied to surrounding areas. For this reason, the positions presented in Figure 2 are between consecutive FBGs to evaluate the force sensitivity variation as a function of the position of applied force. The tests are performed by means of applying a set of calibrated masses, which result in the forces, with a contact cross-sectional region with a 20 mm diameter, such diameter ensures that the force is applied directly on each FBG. The reference measurement of the applied force is made by using the weight with certified calibration (such masses are used for calibration of precision scales). Furthermore, each mass was double checked using another precision scale (Balmak BK-F/FA, Santa Bárbara d’Oeste, Brazil). It is important to mention that these tests are performed with both FBG interrogation units, the first one to acquire the sensitivity of each FBG. Then, using the portable interrogation unit, the tests are repeated to obtain the responses at each filter convolution for each position and force condition, where the sensitivity of each FBG previously acquired can be used for further enhancement of the force distribution detection. It should be noted that the externally applied forces in the characterization tests produce sensor responses equivalent to those induced by GRF during gait. In both cases, the loads act on the same planes and directions and with comparable magnitudes. Accordingly, the term force is used to describe externally applied loads in characterization experiments, whereas GRF refers to forces measured during volunteer gait tests.

Then, the sequential bending and force are applied to the insole from Position 6 to Position 1. This approach is employed to resemble the sequence of foot movement during gait in which the first action is the heel strike followed by a rotation of the foot using the ankle as a pivot. Such movement continues until the heel losses contact with the ground [18]. Prior to gait experiments, the response of each FBG was acquired using the edge-filter interrogation system to establish the expected signal variation during testing. While FBGs are intrinsically sensitive to both strain and temperature, these effects manifest at distinct frequency ranges. Strain signals exhibit rapid fluctuations, necessitating dynamic acquisition rates above 50 Hz, whereas temperature variations are comparatively slow, particularly under controlled laboratory conditions. Consequently, temperature-induced shifts can be effectively suppressed through band-pass filtering.

For the gait tests, 5 volunteers wear the insole positioned on the left foot with a shoe size on the range of 41–43 (Brazilian shoe size) and perform sequential gait cycles at a straight path using the edge filter based FBG interrogation unit, which is not position on the shoe to not affect the natural pattern of gait, where the unit can be positioned on the ankle or on the belt around the waist. The optical fiber connector from the insole is directly connected to the interrogation system, which makes it possible to position the interrogation system at different places around the body, since the optical fiber cable is flexible and compact. It is worth noting that we have the informed consent of the patients for the tests, which was performed following the guidelines of the national health council with the protocols approved by Research Ethics Committee through the National Commission in Research Ethics–CONEP–(Certificate of Presentation for Ethical Appreciation–CAAE: 83936324.3.0000.5542). In these tests, the GRF estimated from the FBG array convolution is analyzed for each step performed with the left foot. Since the force detection capability and accuracy were already characterized in the force characterization tests, the GRF analysis is performed as a function of the curve’s shape, where there is an M-shaped pattern, which is characteristic of the vertical GRF in gait cycles [18]. The curve shape is analyzed for every step and the detection of the stance phase subdivisions are performed, considering the ones presented in [18]. In summary, the subdivisions considered in this analysis are the heel strike (HS) phase when the heel touches the ground; Maximum weight acceptance (MA) when the heel is in full contact with the ground and the entire person’s weight applied; Flat foot (FF) when the whole foot is in contact with the ground and a stable support for the body is achieved; Heel off (HO) when the heel losses contact with the ground; Toe off (TO) phase when the toe losses the contact with the ground, i.e., the swing phase starts.

## 3. Results and Discussions

**Figure 4:**
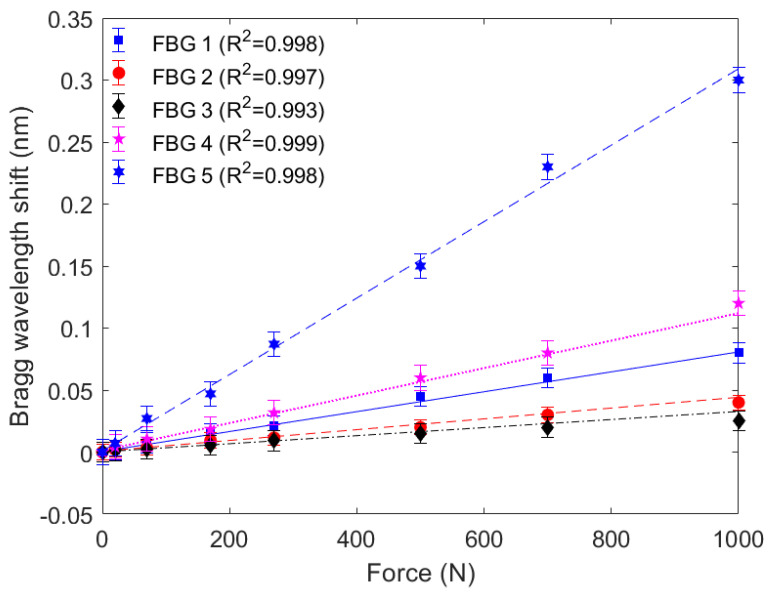
Bragg wavelength shift as a function of the applied force for each FBG.

As the first characterization of the proposed device, the force characterization for each FBG is presented in Figure 4, where it is possible to observe different sensitivities between the FBGs. These differences can be related to their positioning inside the PTFE tube, since it is not possible obtain a micrometer-precision alignment of the optical fiber inside the tube. In this case, the highest sensitivity was obtained in FBG 5 (0.3 pm/N), whereas the highest determination coefficient (R^2^) was obtained in FBG 4 (0.999), which indicates high linearity of the sensor system. Considering the responses of all FBGs, a mean sensitivity of 0.11 ± 0.10 pm/N was obtained with a mean R^2^ of 0.997. Furthermore, the error bars presented in the responses of Figure 4 are the standard deviations of the 3 test cycles for each force at each FBG. The test cycles include loading and unloading of the forces, where the standard deviation presented in Figure 4 also accounts for the hysteresis of each FBG. In this case, the mean hysteresis of the sensor system is around 7% and remains below 10% for all FBGs, where the smallest hysteresis of 2.1% was found in FBG5, whereas the highest hysteresis found is around 9.8%, obtained in FBG 3. Such small hysteresis makes the sensor suitable for dynamic force measurements.

**Figure 5:**
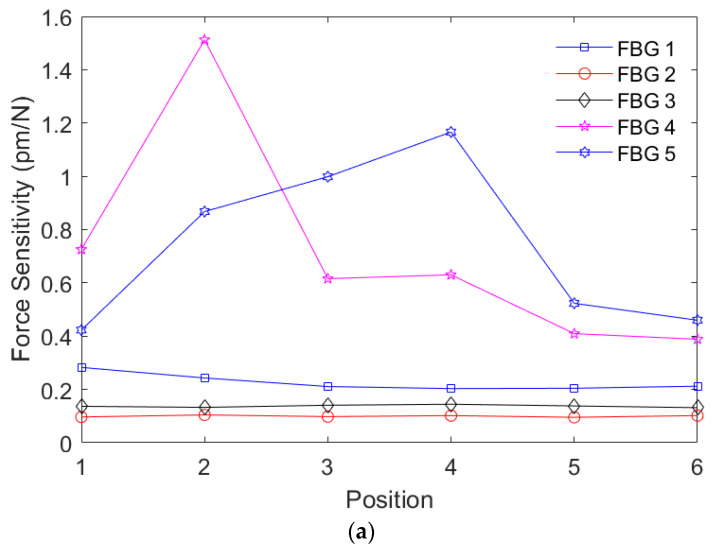

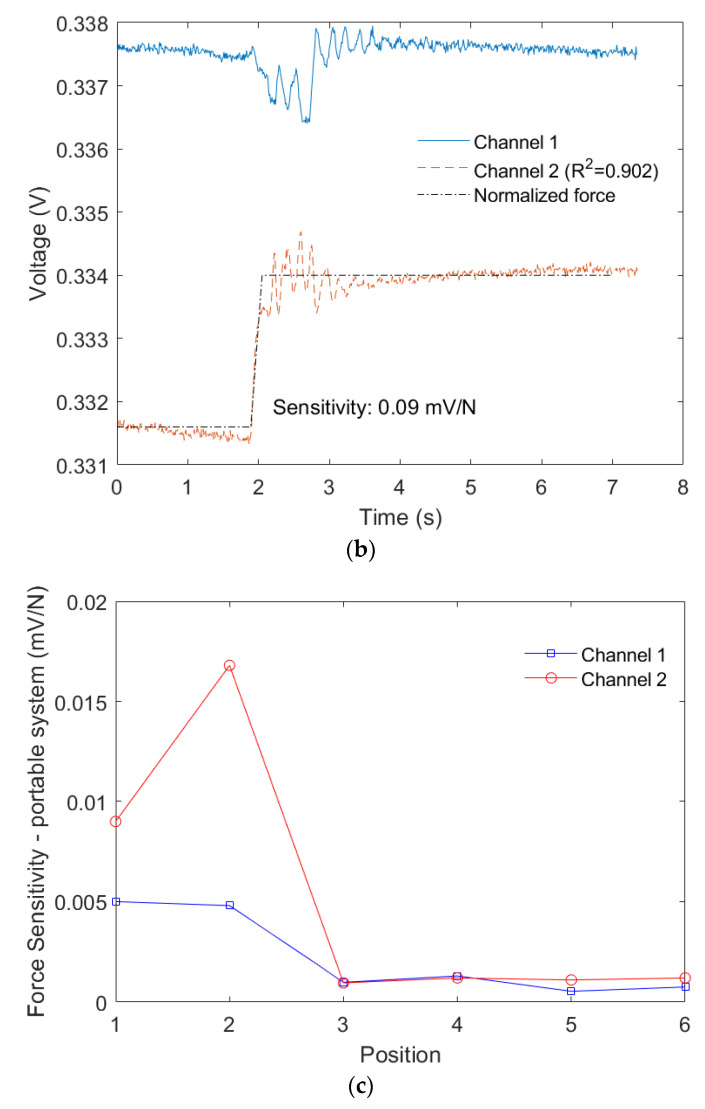
(**a**) FBG sensitivities as a function of the force position applied at each region. (**b**) Optical power variation as a function of time for both edge filters for the forces applied at Position 2. (**c**) Optical power variation in the FBG-embedded insole with the edge filter interrogation approach for each position.

Thereafter, the distributed force characterization is performed by means of applying the forces (in the same interval of the force characterization) at predefined regions (the Positions 1 to 6 presented in Figure 2). In this scenario, the results presented in Figure 5a indicate the sensitivities of each FBG for the forces applied at all predefined regions. In general, the results in Figure 5a show a higher sensitivity of the FBG closer to the position of the force application, since there is a higher wavelength shift for the applied force when the force is applied closer to the grating with maximum sensitivity when the force is directly applied on the FBG region. The reason for this behavior is related to the strain transmission to the FBG, since the applied force leads to an axial strain on the fiber, which leads to the Bragg wavelength shift proportional to the strain on the grating region. Thus, when the distance between the force application region and the grating position increases, there is a smaller strain transmitted to the FBG region, which leads to smaller wavelength shift as a function of the force for this specific position, resulting in a smaller force sensitivity.

It is important to mention that such results are obtained using the bulk FBG interrogator for the characterization of each FBG. In contrast, the results in Figure 5b present the same test, but using the portable interrogation unit, where the results are related to the optical power variation (proportional to the voltage variation for each TIA) as a function of time for the tests in Position 2. It is possible to observe a higher variation in Channel 2. Such higher variation is due to the proximity of the FBGs aligned with the edge filter of Channel 2.

In order to evaluate the force variation at different positions for the FBG-embedded insole with the portable FBG interrogation unit, Figure 5c shows the optical power variation in Channels 1 and 2 as a function of the force for each force application region. The results indicated a variation in each channel responses as a function of both force amplitude and application position.

**Figure 6:**
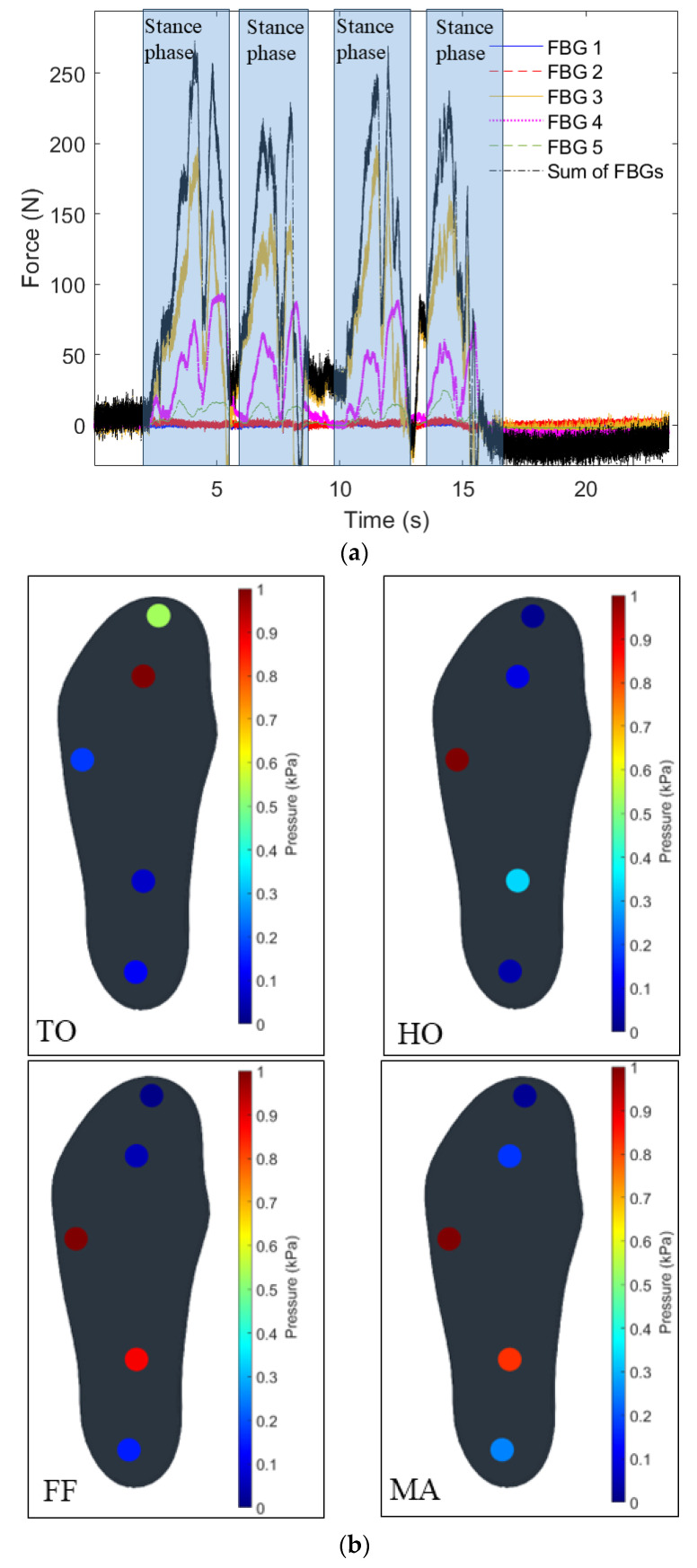

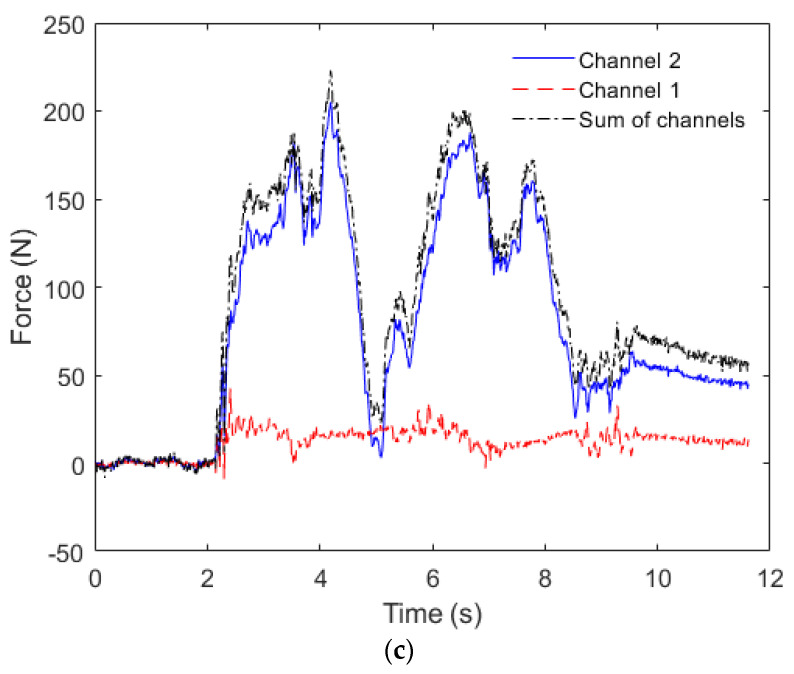
(**a**) Force estimation of each FBG and their sum for the sequential force application. (**b**) Normalized pressure distribution using the proposed sensor system at different predefined gait phases of TO, HO, FF, MA. (**c**) Force estimation through the optical power variation measured at each channel of the portable FBG interrogator.

The force characterization results indicated the sensitivities for the GRF analysis, where the first test is the sequential force application from the heel to the toes of the insole (i.e., from Position 6 to Position 1) to emulate the sequence commonly occurred during gait. In this test, there is also the analysis of each FBG using the bulk FBG interrogation unit and the comparison with the edge filter approach. Figure 6a shows the force estimation for each FBG of the array as a function of the sequential force application, where the results also present the sum of the forces measured by each FBG to obtain a curve that resembles the GRF. Furthermore, Figure 6b shows the pressure mapping capabilities of the proposed device, where the normalized pressure is analyzed at 4 different conditions. In this case, the pressure distribution is analyzed at 1 s, 3 s, 5 s and 7 s along the test presented in Figure 6a. The results in Figure 6b indicate the feasibility of pressure mapping using the proposed device, where there is the pressure distribution variation throughout the gait tests.

**Figure 7:**
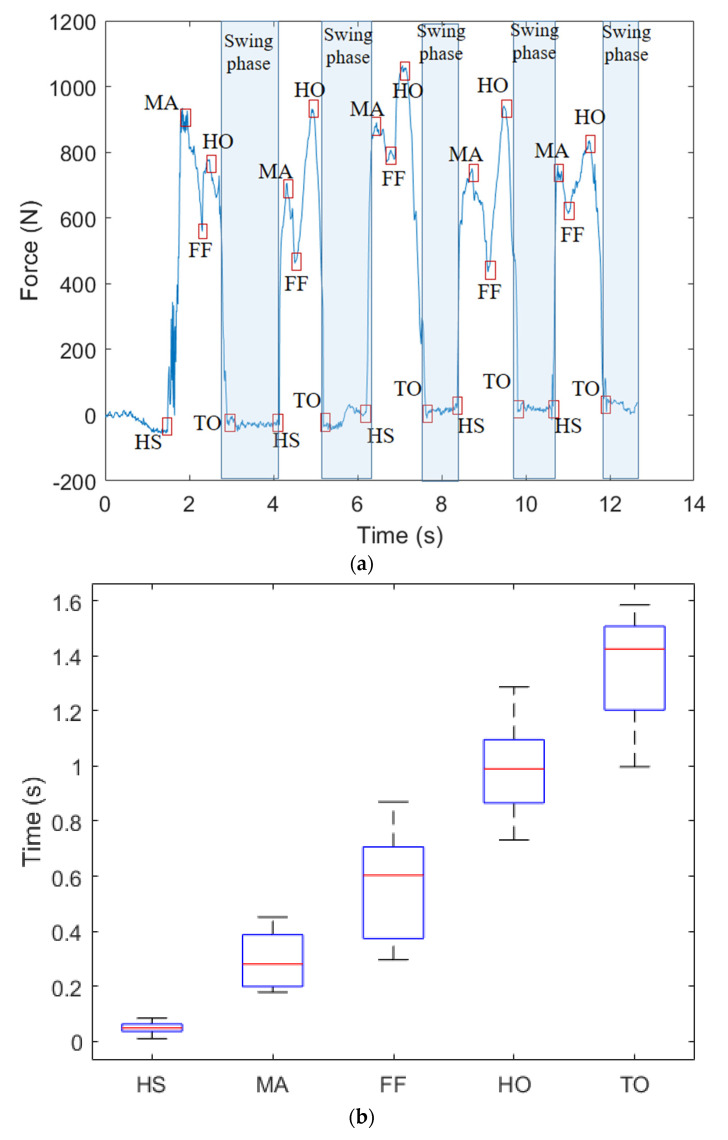

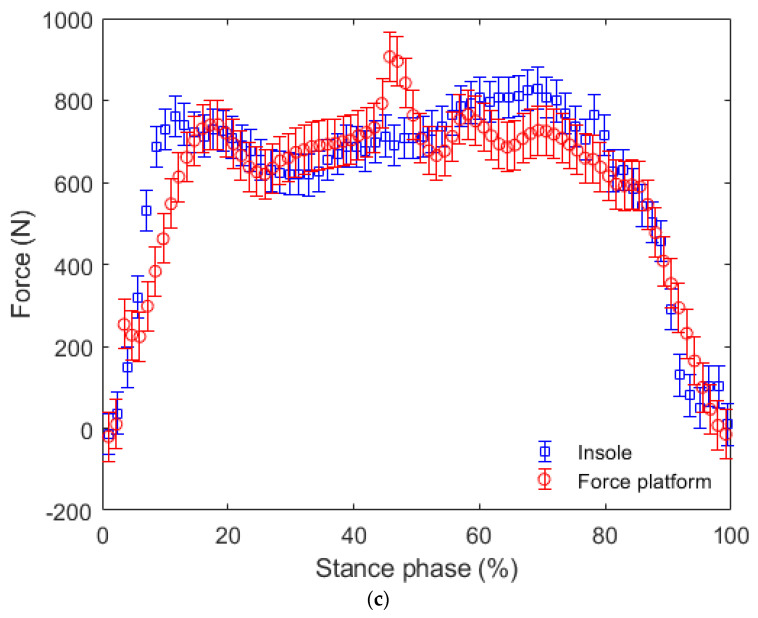
(**a**) GRF estimation of the left foot during gait using the proposed FBG-embedded insole with a portable FBG interrogator. (**b**) Boxplot of the estimated stance phases detected using the proposed fully portable insole. (**c**) Comparison of the proposed sensor system with commercial force platform.

The results considering each FBG (using the bulk FBG interrogator) are compared with the ones of both edge filters from the portable FBG interrogator, where the latter results are presented in Figure 6c. The comparison between both approaches indicated a high correlation between the force estimation from the bulk interrogator and the portable one, which shows the feasibility of the proposed FBG-embedded insole for wearable gait analysis in conjunction with a portable FBG interrogation approach. It is important to mention that some deviations in the results (using both interrogation approaches) can be related to the dynamic behavior of the hosting material (i.e., the TPU material used in 3D printing), since it is a viscoelastic material, which can induce hysteresis in the sensor responses. Such behavior can be compensated using viscoelastic compensation techniques [37], which will be explored in future works. Regarding the results in Figure 6a, FBGs 1 and 2 presented the smallest force variation, which can be related to two reasons. The first one is their positioning on the insole, where there is a smaller force applied during the gait. The second reason is related to the force sensitivity of the individual gratings, since it is not possible to guarantee concentric position of the fiber inside the PTFE tube. In this case, it is possible to have FBGs in different radial positions inside the tube, which directly affect the sensitivity, since the PDMS resin layer is thicker on the FBGs positioned on the bottom of the PTFE tube (i.e., not concentric with the tube). Such higher layers lead to smaller force sensitivity due to the smaller strain transmission with higher layer thickness. Such behavior is also related to the results in Figure 6c, where the results in Channel 1 are related to FBGs 1 and 2, which have smaller force sensitivity, whereas the Channel 2 includes the sum of 3 FBGs (namely FBGs 3, 4 and 5) resulting in higher signal variations not only due to the higher number of gratings for this Channel, but also due to the higher force sensitivities of FBGs 4 and 5. In this case, the portable interrogation system provides the multiplexed response of Figure 6c, where Channel 1 presents the sum of FBGs 1 and 2, whereas Channel 2 provides the responses for FBGs 3, 4 and 5, resulting in the division of the forefoot and midfoot/hindfoot regions. The force applied in such regions (i.e., GRF in the gait) is used to address the divisions of the stance phase, which are further explored in Figure 7a,b.

The results in Figure 6b show the normalized pressure distribution on each FBG for the tests considering each gait phase. In this case, for the TO phase, the FBGs with highest signal variations are the ones on the tip of the insole, i.e., FBGs 4 and 5, whereas the other sensors remain with smaller signal variation. Then, when the HO phase is considered, the highest pressure still on the front part of the insole (since it represents the heel losing contact with the ground), but the highest normalized pressure variation is on FBG 3 (instead of FBG 4 in TO phase). Thereafter, the FF condition is when the whole foot is in contact with the ground, where the highest pressures are applied on the middle region of the insole, represented by the highest pressures measured in FBGs 2 and 3. Finally, in MA phase, there is the load acceptance in the back part of the insole, resulting in variations in FBGs 1 and 2, but with the additional bending of the insole due to the recent heel strike that also translates in higher signal variations in FBG 3.

After the force characterizations and force distribution tests, the volunteers wear the proposed instrumented insole and sequential gait tests are performed. The results of force estimation during the stance phase of the gait are presented in Figure 7a, where it is possible to verify the repeatability of the proposed approach. It is important to mention that a standard deviation of GRF curve is expected since there are natural variations in the gait [36]. However, it is possible to observe that the curve still resembling the M-shaped curve commonly found in GRF. The R^2^ between the sensor response and the M-shaped curve is around 0.904, which indicate a strong correlation between both curves. Furthermore, for the sequential gait cycles also presented in Figure 7a, the subdivisions of the stance phases were identified in all cases, where the HS, MA, FF, HO and TO were identified in accordance with the reference works in the literature [18]. In addition, the swing phase can be estimated from the time at which a small force (ideally zero force) is detected. In this case, a mean swing phase of 41.0 ± 6.5% was obtained, which is in accordance with the swing phase in a health individual [18]. In order to provide a statistical analysis of the results considering all volunteers, Figure 7b shows a boxplot of the detection times of each stance phase subdivision to verify their variance throughout the sequential gait tests, where it is possible to verify the repeatability of the sensor system in which the variations are related to the natural variability of the human gait across different volunteers.

The comparison between the optical fiber sensor system and a gold standard force detection method is performed by means of a comparison between the optical fiber embedded insole and a commercial force platform. In this case, the mean and standard deviations of three consecutive cycles are presented in Figure 7c, where it is possible to verify the high correlation between the FBG insole and the force platform. In this case, an R^2^ higher than 0.9 was found between both approaches, which can be regarded as a high correlation between the sensor systems.

In order to compare the functionalities and the properties of the proposed sensor system, Table 2 presents the comparison between the proposed system and previously reported ones in terms of total weight, relative cost (considering the cost of the reported equipment of each work) and resolution of the sensor systems. In this case, the proposed sensor system is able of providing distributed measurement with higher resolution than the intensity variation sensor system (reported in [28]), but with smaller cost, weight and potability (since it does not need a PC connection) than the other FBG-based insoles (reported in [29,33]). It is important to mention that the resolutions of the systems in Table 2 were estimated from the reported force sensitivities and the wavelength resolution of each acquisition system.

## 4. Conclusions

This paper presented the development and application of an FBG-embedded insole for force distribution and GRF estimation. In this case, a 3D-printed insole is fabricated from a scanned model of a commercial insole in which a 5-FBG array is integrated. The FBGs are characterized as a function of the applied transverse force, where a mean sensitivity of 0.11 ± 0.10 pm/N was obtained considering all FBGs. Moreover, FBG 5 presented the highest sensitivity (0.3 pm/N) for the force distribution analysis at 6 different force application regions. Since commercial FBG interrogators for spectral reconstruction are generally non-portable, a portable FBG interrogator using an edge filter approach is employed, where two filters are used to obtain a broader variation in the FBGs variations. Then, the gait tests were performed, indicating stance and swing phases of 41.0 ± 6.5% and 59 ± 6.5%, respectively, which are within reference values of the literature. In addition, 0.904 R^2^ was found in the correlation analysis of the measured GRF response and the conventional M-shaped curve for the GRF. Therefore, the results indicated the feasibility of the proposed approach for gait analysis and, especially, in GRF and gait phase estimations as a portable option for such analyses that are conventionally restricted to clinical environments. It is also worth mentioning that the linearity of both channels as a function of the force amplitude and position also leads to the possibility of estimating the center of mass of the user, which will be explored in future works for the integration of the proposed FBG-embedded insole with the control system of a lower limb prosthesis. In addition, tests with a higher number of volunteers with specific health conditions will be explored in future work.

## Figures and Tables

**Table 1:** Comparison of sensing performance of different instrumented insoles.

Work	Measurement Principle	Number of Sensors	Pressure Mapping Capabilities	Sample Frequency	Portable
[28]	Intensity variation	1	No	60 Hz	Yes
[29]	FBG array	6	Yes	960 Hz	No
[33]	FBG array	16	Yes	40 Hz	No
[34]	F-scan	Distributed	Yes	750 Hz	Yes
This work	FBG/edge filters	5	Yes	100 Hz	Yes

**Table 2:** Relative cost, resolution and total weight comparison between different instrumented insoles.

Work	PC Connection	Relative Cost	Resolution	Total Weight
[28]	Not mandatory	~200 USD	~10.0 N	~300 g
[29]	Yes	~20,000USD	~0.5 N	~600 g
[33]	Yes	~4400 USD	~5.0 N	~100 g
This work	Not mandatory	~2500 USD	~8.0 N	~335 g

## Data Availability

The raw data supporting the conclusions of this article will be made available by the authors on request.
